# The efficacy and safety of indobufen in patients with ischemic cardiovascular or cerebrovascular diseases: systematic review and meta-analysis

**DOI:** 10.3389/fcvm.2024.1509010

**Published:** 2025-01-09

**Authors:** Xiaolu Luo, Chenglu Lai, Tielong Chen

**Affiliations:** ^1^ Hangzhou Clinical Medical College, Zhejiang Chinese Medical University, Hangzhou, Zhejiang, China; ^2^ Department of Cardiology, Hangzhou Hospital of Traditional Chinese Medicine Affiliated to Zhejiang Chinese Medical University, Hangzhou, Zhejiang, China

**Keywords:** indobufen, cardiovascular, cerebrovascular, efficacy, meta-analysis

## Abstract

**Objective:**

This meta-analysis aims to evaluate the safety and efficacy of indobufen in the treatment of cardiovascular diseases, cerebrovascular diseases, and thromboembolic disorders. The primary focus is on the incidence of major adverse cardiovascular events (MACE), thrombosis, bleeding events, and adverse reactions. The results are intended to provide a reference for the clinical application of indobufen and suggest directions for further large-scale, multi-center, prospective studies.

**Methods:**

This review follows the Preferred Reporting Items for Systematic Reviews and Meta-Analyses (PRISMA) guidelines. A comprehensive search was conducted in PubMed, Embase, Web of Science, and the Cochrane Library databases to identify all relevant studies on indobufen. Twelve trials, all randomized controlled trials (RCTs), met the inclusion criteria. The results were presented as risk ratios (RR) with 95% confidence intervals (CI), and meta-analysis was performed using RevMan 5.3 and Stata 18.0 software.

**Results:**

The meta-analysis included 12 randomized controlled trials. Regarding safety, indobufen showed superior clinical outcomes compared to other antiplatelet agents regarding bleeding events, gastrointestinal adverse reactions, and overall adverse reactions, with these differences being more pronounced in cardiovascular diseases. However, the effects of both treatments on efficacy outcomes, including MACE, myocardial infarction, angina, mortality, and thrombotic events, were similar. For stroke events, particularly in patients with cerebrovascular diseases, the use of indobufen was associated with some risk.

**Conclusion:**

Indobufen is associated with a lower risk of adverse reactions and bleeding, making it a viable option for patients at risk of bleeding or adverse effects, particularly in those with cardiovascular diseases. However, compared to anticoagulants such as aspirin and clopidogrel, indobufen has a shorter history of use, and its evidence base is relatively limited, highlighting the need for further research. Currently, indobufen is widely used in secondary cardiovascular and cerebrovascular prevention and provides some guidance for antiplatelet therapy in patients with gastrointestinal discomfort or bleeding risk. However, due to the potential risks in MACE, stroke, and other events, further clinical trials are needed to assess the clinical applicability of indobufen.

**Systematic Review Registration:**

https://www.crd.york.ac.uk/, identifier (CRD42024587938).

## 1 Introduction

Indobufen, a non-steroidal anti-inflammatory drug (NSAID), has demonstrated significant antiplatelet aggregation effects, making it widely used to prevent and treat cardiovascular diseases. Cardiovascular diseases, one of the leading causes of death and disability worldwide, are steadily increasing in prevalence. According to statistics from the World Health Organization (WHO), ischemic heart disease and stroke rank first among the top ten causes of death worldwide. Cardiovascular and cerebrovascular diseases cause approximately 14 million deaths annually, accounting for 23% of the total global mortality (1). With the aging population and lifestyle changes, the incidence of cardiovascular diseases is on the rise, highlighting the critical need for effective antithrombotic and antiplatelet therapies.

The formation of intravascular thrombosis is the primary cause of cardiovascular and cerebrovascular events (2). Arterial thrombosis primarily comprises platelets and forms at sites of atherosclerotic vascular injury under increased shear stress and disturbed blood flow (3). Antiplatelet drugs, which reduce thrombosis by inhibiting platelet aggregation, play a key role in significantly reducing the incidence of cardiovascular events and are an essential therapeutic approach for treating cardiovascular diseases. Indobufen exerts its antiplatelet effects by inhibiting cyclooxygenase activity (COX-1 and COX-2), thereby reducing the synthesis of platelet aggregation-promoting substances (4). In recent years, there have been an increasing number of studies assessing the effectiveness and safety of indobufen in treating cardiovascular conditions. However, results from various studies have been inconsistent; some demonstrate significant efficacy in preventing cardiovascular events (5), while others fail to confirm its superiority (6). Hence, a systematic evaluation of indobufen's role in cardiovascular disease treatment is crucial.

This study aims to assess the efficacy and safety of indobufen in treating cardiovascular diseases through a systematic review and meta-analysis systematic review of existing literature, thus exploring its potential clinical value. This provides a basis for further clinical research and offers valuable guidance for treating patients with cardiovascular diseases.

## 2 Methods

According to PRISMA (Preferred Reporting Items for Systematic Reviews and Meta-Analyses), the protocol for this meta-analysis was registered on PROSPERO (Registration No: CRD42024587938) (7).

### 2.1 Inclusion criteria

Study Population: Adult patients with cardiovascular or thromboembolic diseases. Intervention: With no dosage or treatment duration restrictions, Indobufen can be used as a single treatment or in combination with other standard treatments. Control: A placebo or another positive control drug, alone or in combination with other standard treatments, without restrictions on dosage or duration. Outcome Measures: Efficacy outcomes including MACE (major adverse cardiovascular events), stroke, myocardial infarction, thrombosis, and mortality, and safety outcomes such as adverse reactions and bleeding. Study Design: Randomized controlled trials (RCTs).

### 2.2 Exclusion criteria

Studies such as conference abstracts, reviews, animal experiments, or republished research; studies where the treatment group does not use indobufen; and studies lacking a control group will be excluded.

### 2.3 Search strategy

One reviewer conducted a computer-based search of the PubMed, Embase, The Cochrane Library, and Web of Science databases using the keywords “indobufen”, “cardiovascular diseases” ,“cerebrovascular diseases”, “thromboembolic diseases”, and “randomized controlled trials”. The search strategy combined both MeSH terms and free-text terms. The search covered the period from establishing each database until September 2, 2024 (See the Supplementary Material for details).

### 2.4 Study selection

Two reviewers independently screened the literature based on the inclusion and exclusion criteria. The full texts were downloaded and thoroughly reviewed for studies meeting the criteria, followed by a second screening and cross-checking. Data were then independently extracted by the two reviewers according to a pre-designed data extraction form, including the essential characteristics of the included studies and their results. Based on the Cochrane Risk of Bias tool, the two reviewers independently assessed the methodological quality of the included studies.

### 2.5 Assessment of risk of bias and quality of evidence

Based on Cochrane risk-of-bias criteria, two reviewers (XL and CL) independently assessed the quality of all included trials. Stata was used to calculate the risk of bias.

### 2.6 Data analysis

Analyses were conducted using Review Manager 5.3 and Stata 18.0 software. A heterogeneity test was performed on the data before the meta-analysis. If the studies had no significant heterogeneity (*P* > 0.05 and *I*^2^ < 50%), a fixed-effect model was used to weigh and combine the effect sizes. If heterogeneity was present (*P* < 0.05 and *I*^2^ > 50%), studies with significant heterogeneity were excluded from meeting the heterogeneity threshold, after which a fixed-effect model was used to weigh and combine the effect sizes. The results were expressed as relative risk (RR) with 95% confidence intervals (CI). Subgroup analysis was conducted based on the disease type, study country or region, study type, and drug type. Publication bias was assessed using Egger's test, Begg's test, and funnel plot analysis (*P* > 0.05 indicates no publication bias; confidence level: Egger's test > Begg's test > funnel plot). A *P*-value of less than 0.05 was considered statistically significant. Additionally, sensitivity analysis was performed using the “metaninf” tool in Stata software.

## 3 Results

**Figure 1:**
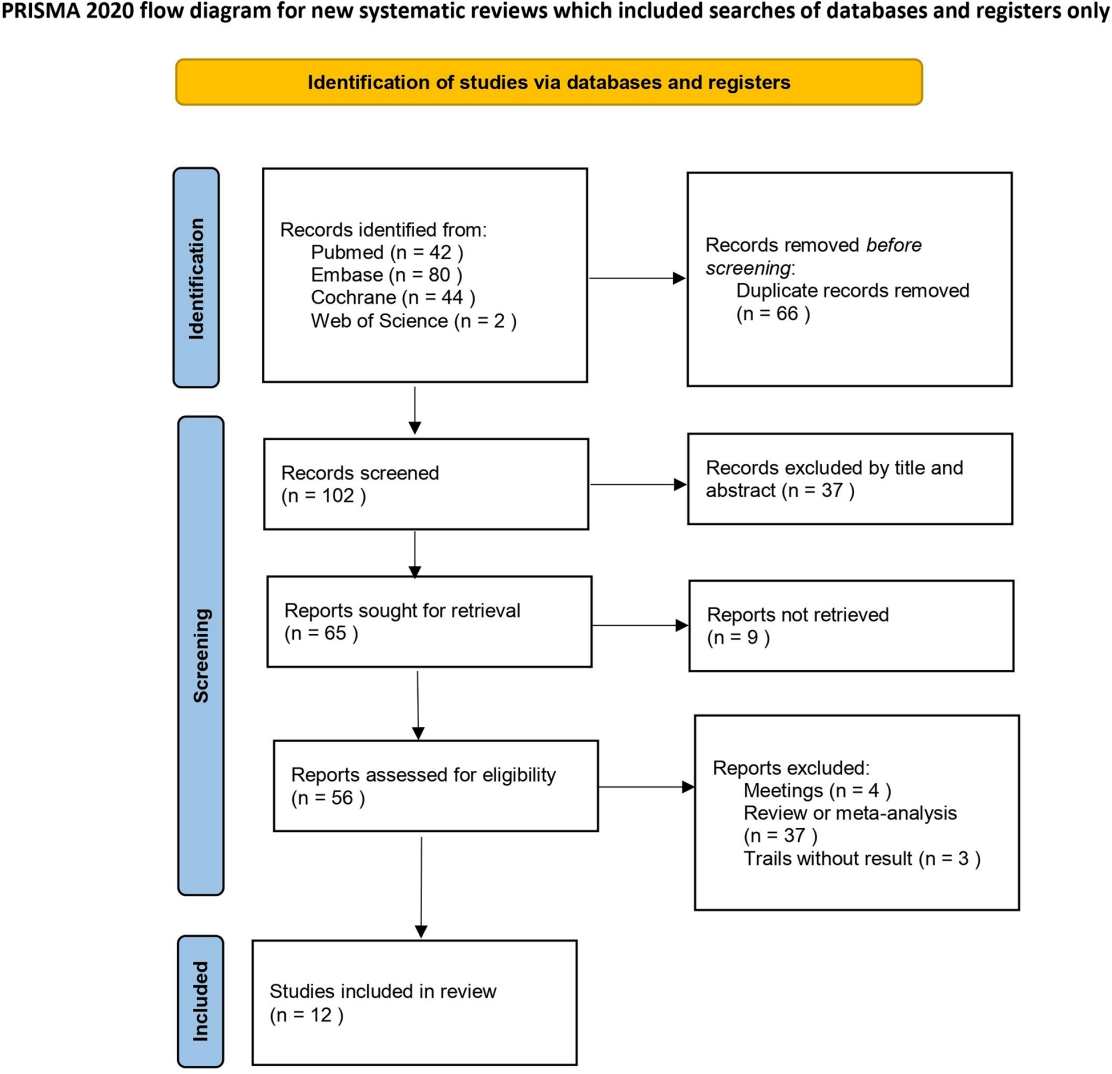
Literature search and screening flow diagram.

After screening the databases, 168 studies were initially identified. After 66 duplicate studies were removed, 37 irrelevant studies were excluded based on their titles and abstracts. Additionally, 37 articles were excluded because they were reviews or meta-analyses. The full texts of 28 articles were further reviewed, resulting in the exclusion of 16 articles. Among these, 3 trials lacked results, 4 were conference abstracts, and 9 studies had unavailable full texts, making data extraction impossible. Ultimately, 12 randomized controlled trials (RCTs) were included in this meta-analysis. A brief description of the literature screening process is shown in Figure 1, while a summary of the key characteristics of the included studies is provided in Table 1.

**Table 1:** Characteristics of included studies.

Study	Country	Participants	Treatment		Design	Age		Sex(F/M)		No. of participants		Outcomes
			Indobufen	Others		Indobufen	Others	Indobufen	Others	Indobufen	Others	
Barilla et al. (8)	Italy	Post-PCI patients allergic to ASA	100 mg Indobufen qd + 75 mg Clopidogrel qd	75 mg Clopidogrel qd	RCT	62.25 ± 11.21	61.05 ± 10.86	6/14	6/15	20	21	①③⑤⑦⑧⑨
Bergamasco et al. (9)	Italy	Patients with stroke	200 mg Indobufen qd/bid	250 mg Ticlopidine qd/bid	RCT	65.7 ± 8.9	65.2 ± 9.0	308/504	299/522	811	821	①②③⑥⑦⑧⑨
Bai et al. (10)	China	Patients received CABG	100 mg Indobufen qd + 75 mg Clopidogrel qd	100 mg Asprin qd + 75 mg Clopidogrel qd	RCT	60.3 ± 6.6	59.7 ± 7.2	19/57	14/62	76	76	①③④⑦⑧⑨
Fornaro et al. (11)	Italy	Chronic NVAF or presenting a potential source of embolism	100 mg Indobufen bid	placebo	RCT			43/55	53	45	98	①②③⑤⑥⑧⑨
Belcaro et al. (12)	Italy	Deep-vein thrombosis	200 mg Indobufen bid	without reatment	RCT	45 ± 11	45.7 ± 12	31/29	32/31	60	63	⑤
Wu et al. (5)	China	Patients undergoing coronary drug -eluting stent implantation	100 mg Indobufen bid + 75 mg Clopidogrel qd	100 mg Asprin qd + 75 mg Clopidogrel qd	RCT	61.0 ± 8.3	61.2 ± 8.4	737/1,521	846/1,447	2,258	2,293	①②③⑥⑦
Yang et al. (13)	China	Patients with coronary atherosclerosis	100 mg Indobufen bid	100 mg Asprin qd	RCT	60.8 ± 10.7	60.7 ± 8.0	13/17	11/21	32	30	⑦⑧⑨
Morocutti et al. (14)	Italy	Patients with NRAF and a recent cerebral ischemic episode	100/200 mg Indobufen bid	Warfarin (to INR 2.0–3.5)	RCT	72.8 ± 8.3	72.2 ± 8.1	252/210	234/220	462	454	①②③⑤⑥⑦⑧⑨
Peters et al. (15)	Holland	Patients with AMI	200 mg Indobufen bid	Acenocoumarol (controlled by thrombotest)	RCT	61.8 ± 13.3	65.5 ± 11.2	19/55	21/55	74	76	①⑤⑥⑦
Rajah et al. (16)	UK	Patients received CABG	200 mg Indobufen bid	300 mg Asprin tid + 75 mg Dipyridamole tid	RCT	54.4 ± 8.3	54.9 ± 8.2	52/349	63/335	399	404	①④⑥⑦⑧⑨
Liu et al. (17)	China	Patients with stroke	100 mg Indobufen bid + 75 mg Clopidogrel qd	100 mg Asprin qd + 75 mg Clopidogrel qd	RCT	60.8 ± 11.5	60.8 ± 12.3	73/20	61/28	89	93	①②⑦
Pan et al. (6)	China	Patients with stroke	100 mg Indobufen bid + Asprin placebo	100 mg Asprin qd + Indobufen placebo	RCT	50.6 ± 70.3	57.71 ± 6.40	964/1,751	957/1,766	2,715	2,723	①②③⑤⑥⑦⑧⑨

Outcomes: ① MACE; ② stroke; ③ myocardial infarction; ④ angina pectoris; ⑤ embolism; ⑥ death; ⑦ bleeding; ⑧ gastrointestinal adverse reaction; ⑨ adverse reaction.

CABG, coronary artery bypass grafting; PCI, percutaneous transluminal coronary intervention; ASA, asprin; NVAF, nonvalvular atrial fibrillation; AMI, acute myocardial infarction; NRAF, nonrheumatic atrial fibrillation.

### 3.1 Risk of bias assessment

**Figure 2:**
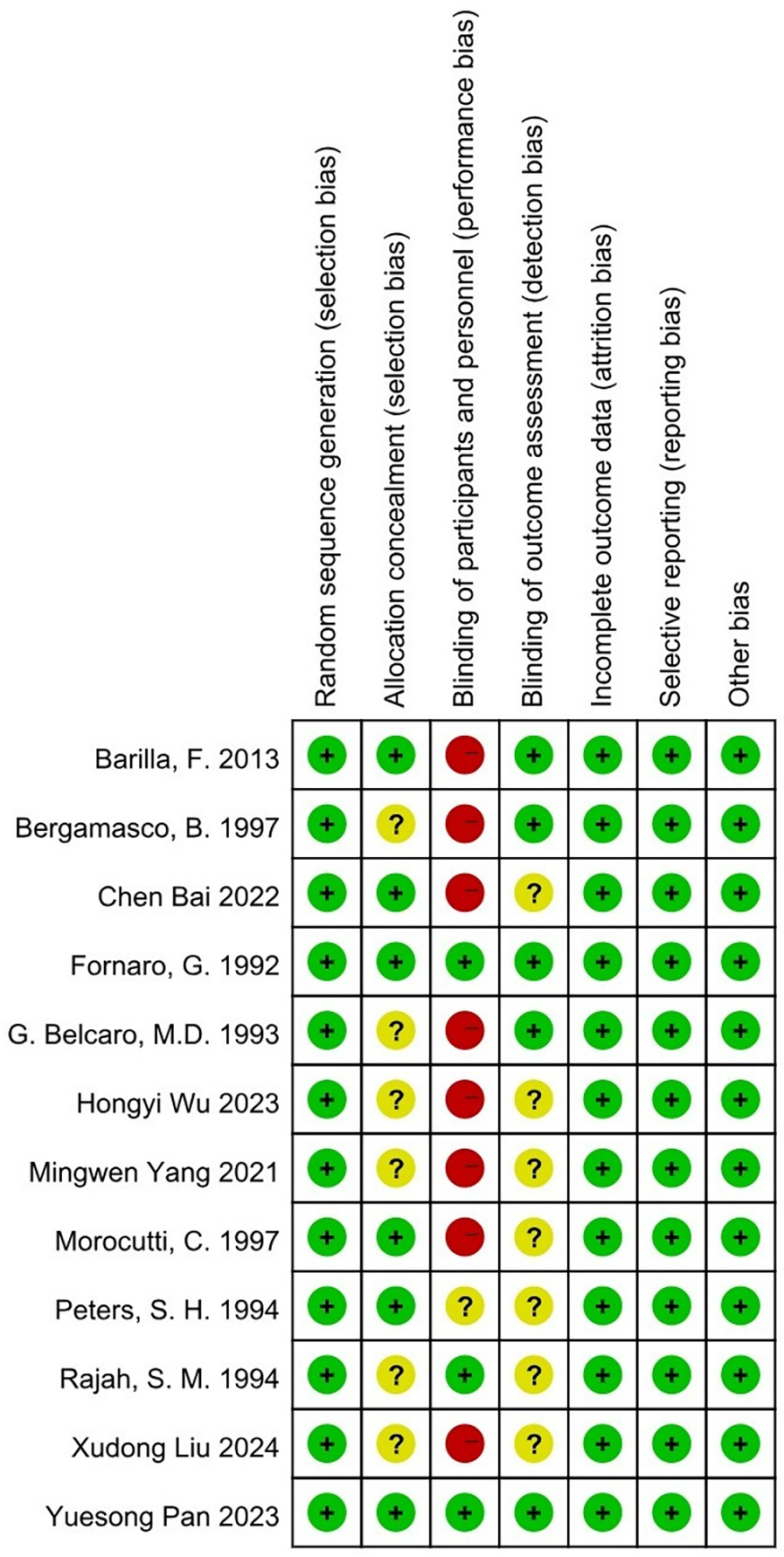
Results of quality evaluation of randomized controlled trials.

An assessment of bias was conducted using Cochrane Collaboration's tool for evaluating randomized controlled trials (RCTs) (18; Figure 2).

### 3.2 MACE (major adverse cardiovascular events)

**Figure 3:**
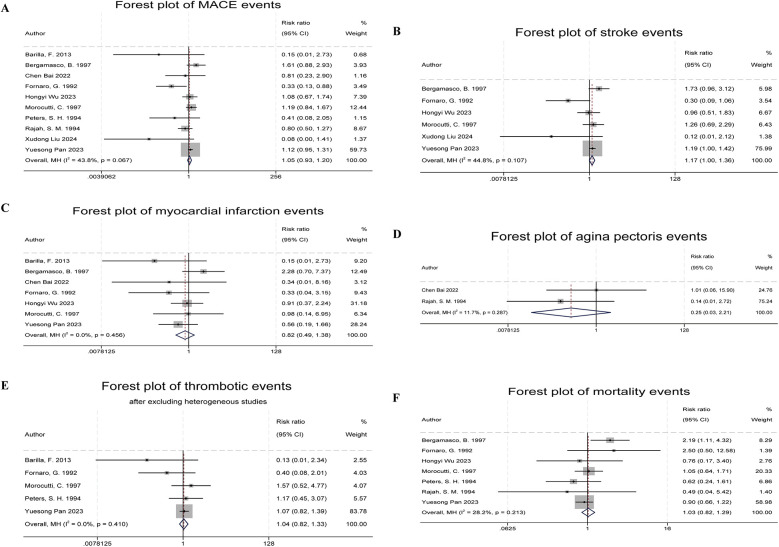
Forest plots of indobufen treatment efficacy evaluations. **(A)** Forest plot of MACE events. **(B)** Forest plot of stroke events. **(C)** Forest plot of myocardial infarction events. **(D)** Forest plot of angina pectoris events. **(E)** Forest plot of thrombotic events. **(F)** Forest plot of mortality events.

Ten studies reported Major Adverse Cardiovascular Events (MACE) as the primary outcome (5, 6, 8–11, 14–17), which primarily included fatal cardiovascular diseases, myocardial infarction, non-fatal stroke, and recurrent angina. The combined results showed no significant difference in MACE incidence between the indobufen and other treatment groups (RR: 1.05, 95% CI: 0.93–1.20, *I*^2^ = 44%, *P* = 0.41; Figure 3A).

### 3.3 Stroke

Six studies reported stroke events (5, 6, 9, 11, 14, 17). The combined results showed a significant difference in stroke event rates between the indobufen and other treatment groups (RR: 1.17, 95% CI: 1.00–1.36, *I*^2^ = 45%, *P* = 0.049; Figure 3B).

### 3.4 Myocardial infarction

Seven studies reported myocardial infarction events (5, 6, 8–11, 14). The combined results showed no significant difference in the incidence of myocardial infarction between the indobufen group and other treatment groups (RR: 0.82, 95% CI: 0.49–1.38, *I*^2^ = 0%, *P* = 0.45; Figure 3C).

### 3.5 Recurrence of angina pectoris

Two studies reported recurrent angina events (10, 16). The combined results showed no significant difference in the incidence of recurrent angina between the indobufen group and other treatment groups (RR: 0.25, 95% CI: 0.03–2.21, *I*^2^ = 12%, *P* = 0.21; Figure 3D).

### 3.6 Thrombotic events

Six studies reported thrombotic events (8, 11, 12, 14, 15, 17). After heterogeneity testing (*I*^2^ = 58.5%, *Q* test *P* = 0.03), the results indicated statistical heterogeneity between the included studies. After excluding the study by Belcaro et al. (12), which had a significant impact on heterogeneity, the combined results showed no significant difference in the incidence of thrombotic events between the indobufen group and other treatment groups (RR: 1.04, 95% CI: 0.82–1.33, *I*^2^ = 0%, *P* = 0.74; Figure 3E).

### 3.7 Death

Seven studies reported mortality events (5, 6, 9, 11, 14–16). The combined results showed no significant difference in the incidence of mortality between the indobufen group and other treatment groups (RR: 1.03, 95% CI: 0.82–1.29, *I*^2^ = 28%, *P* = 0.80; Figure 3F).

### 3.8 Bleeding events

**Figure 4:**
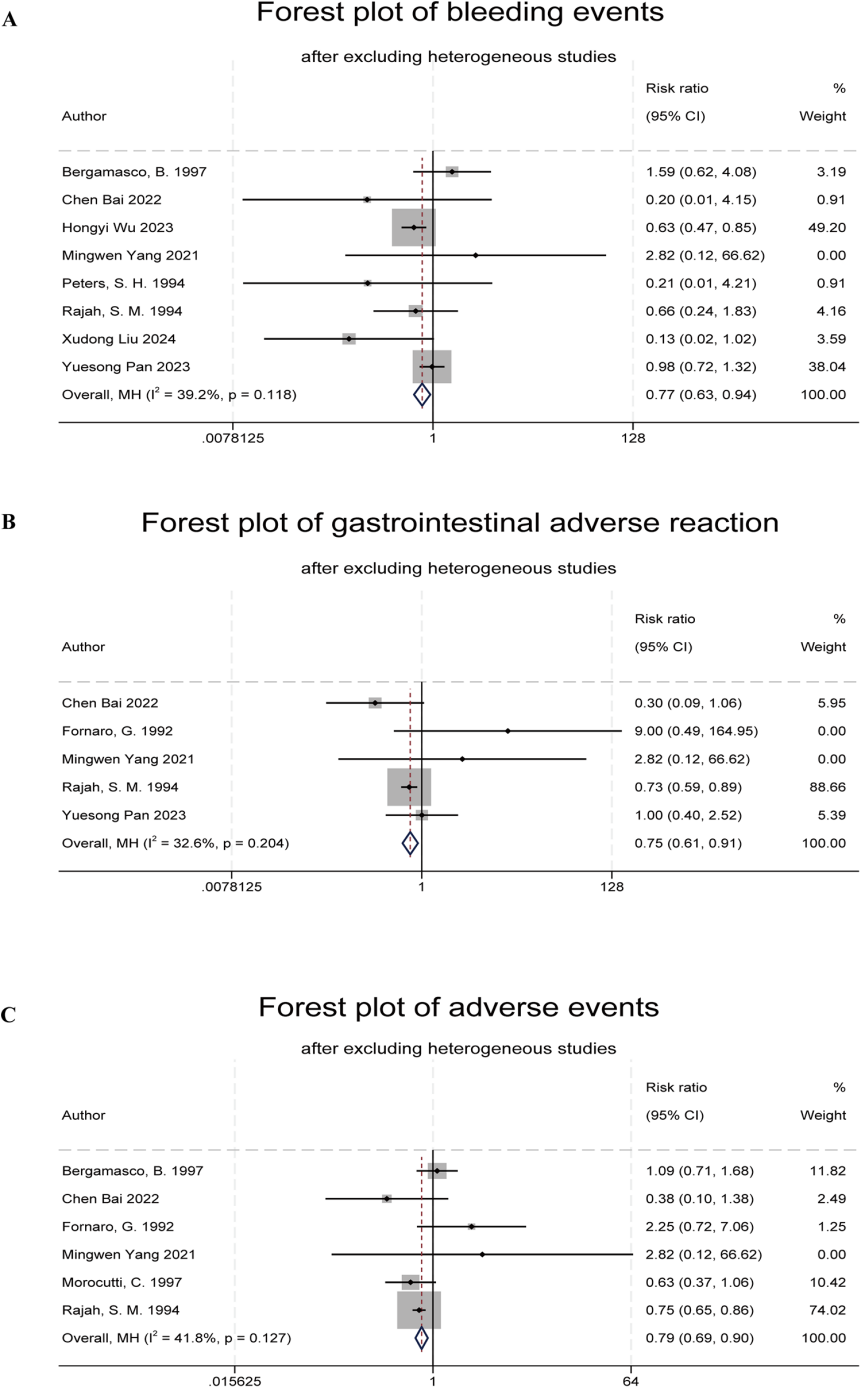
Forest plots of indobufen treatment safety evaluations. **(A)** Forest plot of bleeding events. **(B)** Forest plot of gastrointestinal adverse reaction. **(C)** Forest plot of adverse events.

Ten studies reported bleeding events (5, 6, 8–10, 13–17). One study was excluded due to a lack of event occurrence (8). Heterogeneity testing showed that the study by Morocutti et al. (14) had a significant impact on heterogeneity (*I*^2^ = 62%, *Q* test *P* = 0.008). After excluding this study, the combined results showed a significant difference in bleeding event rates between the indobufen group and other treatment groups (RR: 0.77, 95% CI: 0.63–0.94, *I*^2^ = 39%, *P* = 0.01; Figure 4A).

### 3.9 Gastrointestinal adverse reactions

Six studies reported gastrointestinal adverse reactions (6, 9–11, 13, 14, 16). One study was excluded due to a lack of event occurrence (12). Heterogeneity testing (*I*^2^ = 55.6%, *Q* test *P* = 0.05) indicated statistical heterogeneity. After excluding the study by Bergamasco et al. (9), the combined results showed a significant difference in the overall adverse reaction rates between the indobufen group and other treatment groups (RR: 0.75, 95% CI: 0.61–0.91, *I*^2^ = 33%, *P* = 0.004; Figure 4B).

### 3.10 Total adverse reaction events

Eight studies reported total adverse reaction events (6, 8–11, 13, 14, 16), which included gastrointestinal adverse reactions, dizziness, rash, allergic reactions, and anemia. One study was excluded due to a lack of event occurrence (7). Heterogeneity testing (*I*^2^ = 67%, *Q* test *P* = 0.006) indicated statistical heterogeneity. After excluding the study by Yuesong Pan et al. (6), the combined results showed a significant difference in the total adverse reaction rates between the indobufen group and other treatment groups (RR: 0.79, 95% CI: 0.69–0.90, *I*^2^ = 42%, *P* = 0.0004; Figure 4C).

### 3.11 Subgroup analysis

#### 3.11.1 Subgroup analysis of efficacy evaluation

The subgroup analysis for cerebrovascular diseases indicated a significant difference in stroke event rates between the indobufen and other treatment groups (RR: 1.22, 95% CI: 1.04–1.43, *I*^2^ = 23%, *P* = 0.02). No meaningful results were found in the other subgroup analyses (Supplementary Tables S1–S4).

#### 3.11.2 Subgroup analysis of safety evaluation

In the subgroup analysis of cardiovascular diseases, significant differences were found in the bleeding events (RR: 0.62, 95% CI: 0.47–0.83, *I*^2^ = 0%, *P* = 0.001), gastrointestinal adverse reactions (RR: 0.73, 95% CI: 0.60–0.90, *I*^2^ = 45%, *P* = 0.002), and total adverse reaction events (RR: 0.76, 95% CI: 0.67–0.88, *I*^2^ = 43%, *P* = 0.0001) between the indobufen group and other treatment groups. Additionally, in the subgroup analysis based on the treatment drug, indobufen showed a more favorable effect compared to aspirin and clopidogrel for bleeding events and gastrointestinal adverse reactions (RR: 0.75, 95% CI: 0.61–0.92, *I*^2^ = 41%, *P* = 0.0006; RR: 0.72, 95% CI: 0.59–0.88, *I*^2^ = 1%, *P* = 0.001). No meaningful results were found in the other subgroup analyses (Supplementary Tables S1–S4).

### 3.12 Sensitivity analysis

Heterogeneity tests were conducted when synthesizing effect sizes for each group. If the heterogeneity test indicated statistical significance, studies that contributed substantially to the heterogeneity were excluded. In the final pooled results, heterogeneity was within acceptable limits. Sensitivity analyses were also performed, and the results remained stable (Supplementary Figures S1–S9).

### 3.13 Publication bias

Publication bias was tested for each outcome by performing Egger's test, Begg's test, and funnel plot analysis (Supplementary Figures S10–S18). Both Egger's and Begg's tests showed *P* > 0.05, indicating no significant publication bias for the studies included in the analysis of each outcome.

## 4 Discussion

Based on meta-analysis, this study systematically evaluated the efficacy and safety of indobufen in treating cardiovascular diseases, cerebrovascular diseases, and thromboembolic diseases. Through this meta-analysis, we confirmed that indobufen demonstrates better clinical outcomes than other antiplatelet drugs in safety endpoints, including bleeding, gastrointestinal, and overall adverse reactions. Specifically, subgroup analyses of monotherapy or combination therapy with aspirin or clopidogrel, as well as cardiovascular disease subgroup analysis, revealed that indobufen had a significant advantage in bleeding events and gastrointestinal adverse reactions, which may be attributed to the reversible antiplatelet effect of indobufen. Regarding efficacy, no significant clinical superiority was observed for indobufen in MACE, myocardial infarction, angina, thrombosis, and mortality events compared to other antiplatelet therapies. However, in the case of stroke events, especially among cerebrovascular disease patients, indobufen demonstrated some risks.

Additionally, heterogeneity issues were of considerable concern in this study. Although studies with significant heterogeneity were excluded in the combined analysis, factors such as study design differences, participants' baseline characteristics, and drug dosages may still influence the results. The subgroup analyses in this study showed that heterogeneity in thrombotic events, gastrointestinal adverse reactions, and total adverse reaction outcomes were primarily caused by inconsistent disease types among the included studies. No significant heterogeneity was found in the outcomes mentioned above after performing subgroup analyses based on different disease types (Supplementary Figures S19–S22). For bleeding events, heterogeneity caused by the study by Morocutti et al. (14) and publication bias visible in the funnel plot were further explored. We speculated that the close monitoring of patients in this study during follow-up may have kept the relevant indicators within optimal ranges, which could explain why the original data from this study may not reflect the most realistic research situation compared to other studies.

Although no other meaningful results could be obtained in this study apart from the findings mentioned above, several interesting issues were observed. In cerebrovascular diseases, indobufen treatment may lead to higher risks of thrombosis and gastrointestinal adverse reactions. Further analysis of the included studies suggests that the high risk could be attributed to the few included studies (only two), which may have led to biased results and cannot be generalized. Furthermore, in subgroup analyses of cerebrovascular diseases and Asian patients, indobufen exhibited a higher risk in MACE events and stroke events, which is consistent with the conclusions from the study by Morocutti et al. (14). However, no statistical differences were found between the treatment groups after adjusting for prognostic factors. Multivariable analysis revealed that these factors were female sex, history of stroke, and myocardial infarction at admission (14). It is also well-known that antiplatelet drugs may show low responsiveness in certain diseases or populations due to metabolic pathways or genetic effects, such as the CYP2C19 enzyme for clopidogrel metabolism and the COX-1 gene and P2Y12 gene polymorphisms for aspirin metabolism (19–23). Due to variations in drug dosages, potential drug synergistic effects, the polymorphisms of the genes mentioned above, and the larger weight of findings from studies with large sample sizes, the higher risk associated with indobufen may have been influenced.

Our meta-analysis confirmed that indobufen has certain advantages for patients with gastrointestinal dysfunction or aspirin intolerance in cardiovascular diseases, consistent with expert consensus in clinical guidelines (19). Previous clinical studies, such as OPTION (indobufen or aspirin with clopidogrel for coronary artery drug-eluting stent implantation) and INSURE (indobufen vs. aspirin in acute ischemic stroke), large multi-center clinical trials comparing indobufen and aspirin, reported similar results to our meta-analysis. Indobufen demonstrated advantages in bleeding outcomes compared to other antiplatelet drugs, but for preventing recurrence in cerebrovascular diseases, especially in stroke patients, indobufen was not superior to aspirin. Moreover, a series of previous systematic reviews evaluating the efficacy and safety of indobufen have also pointed out that its effectiveness is significantly lower than other antiplatelet therapies in patients with gastrointestinal discomfort or those at risk of bleeding (24–26).

This meta-analysis comprehensively analyzed the efficacy and safety of indobufen in cardiovascular and cerebrovascular diseases, as well as thromboembolic diseases, and conducted subgroup analyses with possible explanations for significant results. However, due to differences in study designs, such as varied inclusion and exclusion criteria, baseline characteristics of patient populations, and differences in the diseases studied [e.g., coronary artery bypass grafting (CABG), percutaneous coronary intervention (PCI), and non-rheumatic atrial fibrillation (AF)], as well as time spans between studies (from 1993 to 2024). Inconsistent definitions of outcome assessment, these factors could have influenced the final results of the meta-analysis. Nevertheless, it cannot be denied that indobufen shows advantages compared to other antiplatelet drugs in safety outcomes.

## 5 Limitation

Several limitations remain in this study. Firstly, the open-label design of some randomized trials may potentially influence the outcomes and introduce bias into the results. Additionally, Different studies used various metrics to assess treatment efficacy, and the statistical forms of these metrics were inconsistent across studies, such as the thromboxane B2 (TXB2) levels in plasma and urine, making it difficult to compare the outcomes of these studies. Therefore, the research focuses only on safety indicators, such as bleeding events and adverse reactions, as well as efficacy indicators, such as cardiovascular events and stroke.

Third, the included studies had inconsistent dosing regimens and insufficient studies with the same dosing protocols. This inconsistency in dosing regimens limits the interpretation of differences between the two groups in the final pooled analysis and introduces bias. In particular, the limited number of studies related to thromboembolic diseases included in this research made it difficult to evaluate the efficacy and safety of indobufen in treating thromboembolic diseases. Fourth, the studies from Asian countries included in this research were limited to China. While these studies provide valuable references for indobufen use in China and other Asian countries, future research should involve more countries and ethnic groups to obtain better results.

## 6 Conclusion

There are several antiplatelet drugs currently available in clinical practice, including aspirin, clopidogrel, and warfarin. Indobufen, as a secondary medication for preventing cardiovascular and cerebrovascular diseases, exhibits high selectivity and reversible binding to its receptor (27), offering certain advantages over other anticoagulants. However, compared to other anticoagulants, indobufen is used for a shorter duration, and there are fewer large-scale clinical studies, which limits the strength of the available evidence. Additionally, the increased risk of MACE and stroke observed in the indobufen group in this study suggests that, in specific conditions such as cerebrovascular diseases, these risks may be more pronounced. In conclusion, indobufen may be a suitable choice for certain populations. Its low association with bleeding events and adverse reactions provides some guidance for clinical antiplatelet therapy in patients at potential risk of bleeding or adverse reactions. Nevertheless, compared to widely recognized antiplatelet drugs like aspirin and clopidogrel, indobufen still shows certain limitations in clinical efficacy. Therefore, using indobufen should be based on carefully assessing its effectiveness and safety with individualized treatment decisions. Further research focusing on different disease types and populations will be crucial for better defining the indications and scope of use for indobufen in future clinical practice.

## Data Availability

The original contributions presented in the study are included in the article/Supplementary Material, further inquiries can be directed to the corresponding author.
