# Supplementary material for: The efficacy and safety of indobufen in patients with ischemic cardiovascular or cerebrovascular diseases: systematic review and meta-analysis
Source: Front Cardiovasc Med. 2025 Jan 9;11:1509010. doi: 10.3389/fcvm.2024.1509010 (PMC11754262; doi:10.3389/fcvm.2024.1509010)
Supplement: Supplementary file 1 [file Datasheet1.pdf]

Supplementary Material

1 Supplementary Figures

1.1 Sensitivity analysis

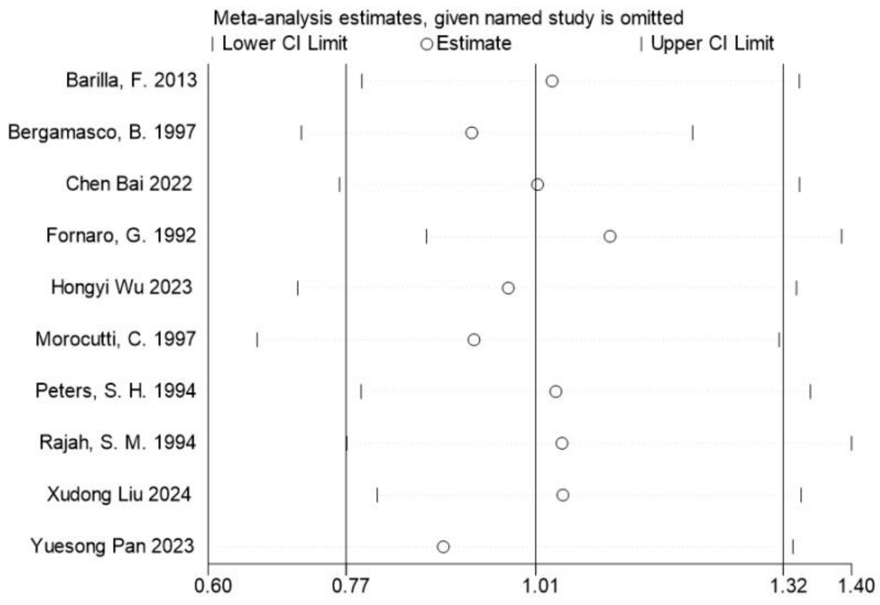

Supplementary Figure 1. Sensitivity analysis of major adverse cardiovascular events (MACE).

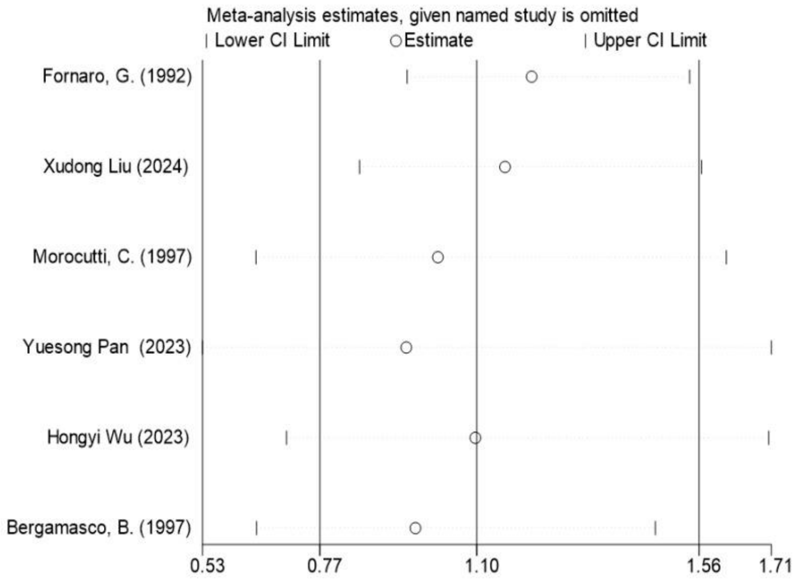

Supplementary Figure 2. Sensitivity analysis of stroke events.

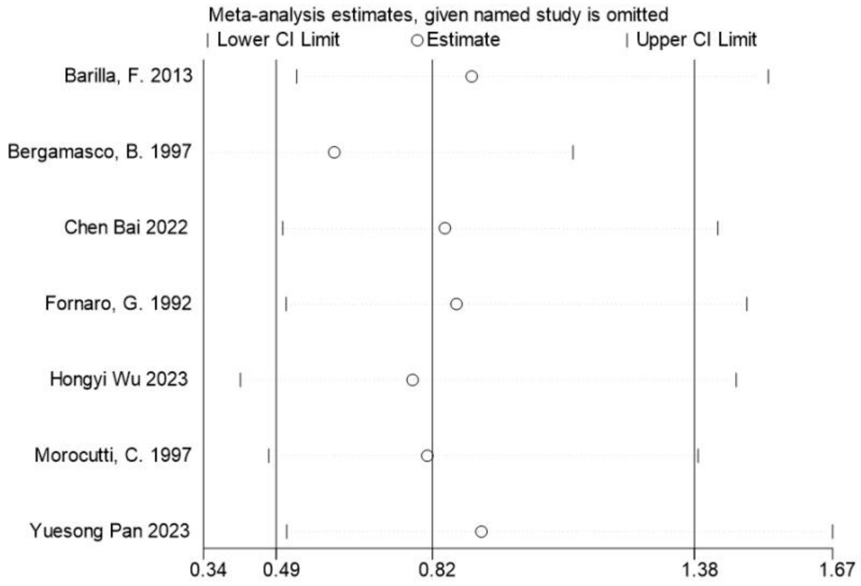

Supplementary Figure 3. Sensitivity analysis of myocardial infarction events.

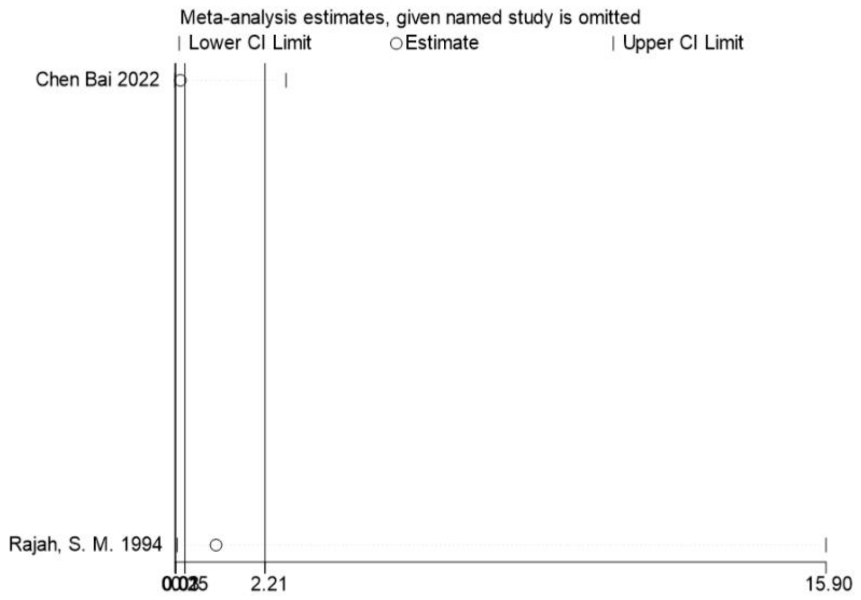

Supplementary Figure 4. Sensitivity analysis of recurrent angina events.

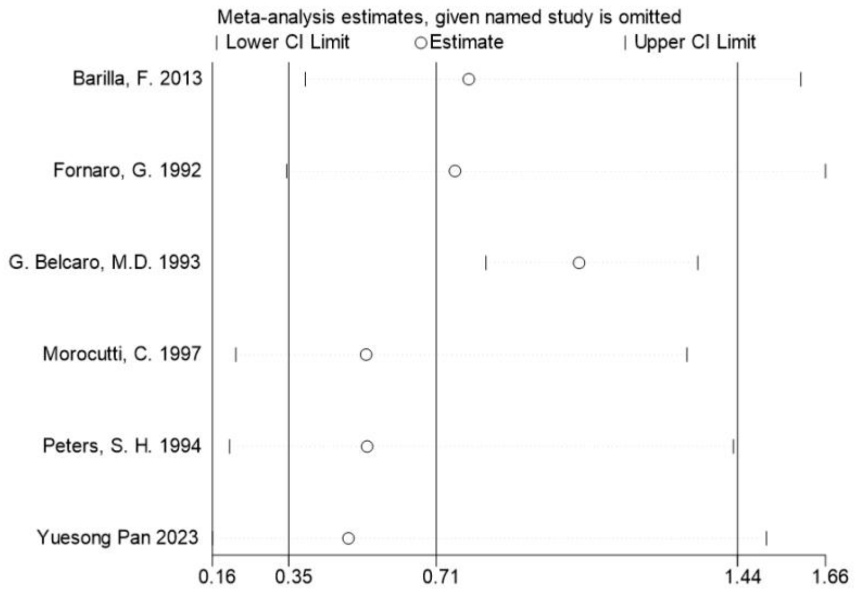

Supplementary Figure 5. Sensitivity analysis of thrombotic events.

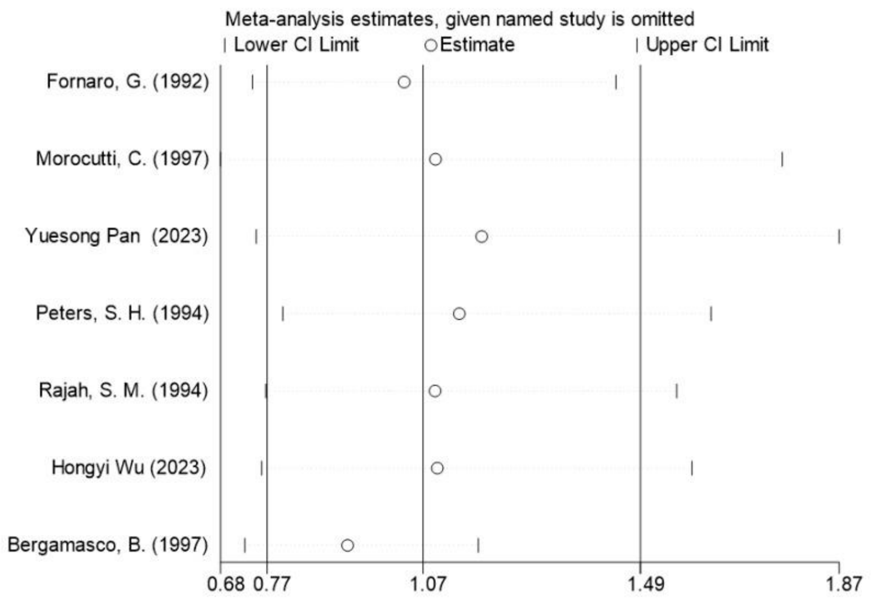

Supplementary Figure 6. Sensitivity analysis of death outcomes.

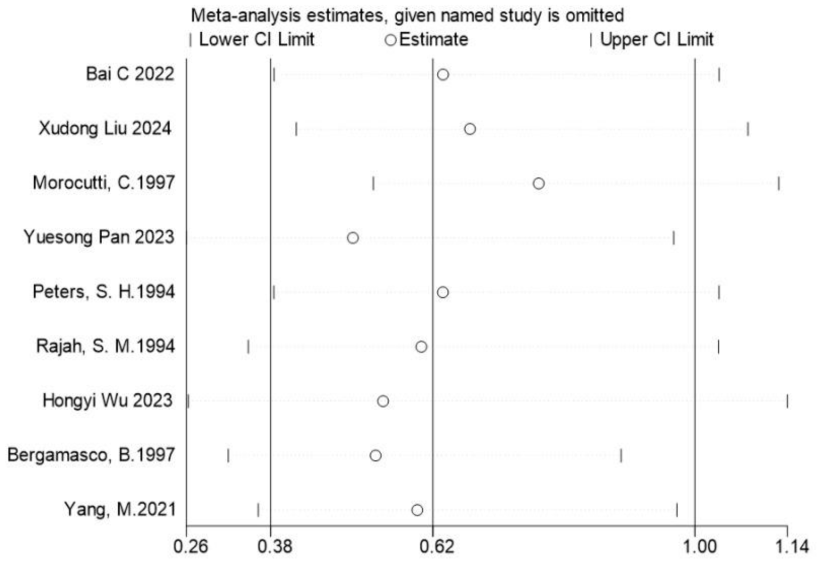

Supplementary Figure 7. Sensitivity analysis of bleeding events.

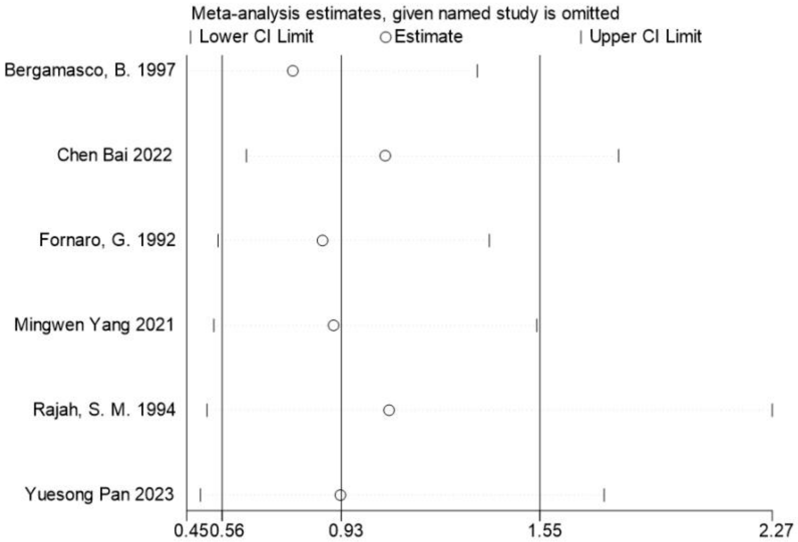

Supplementary Figure 8. Sensitivity analysis of gastrointestinal adverse events.

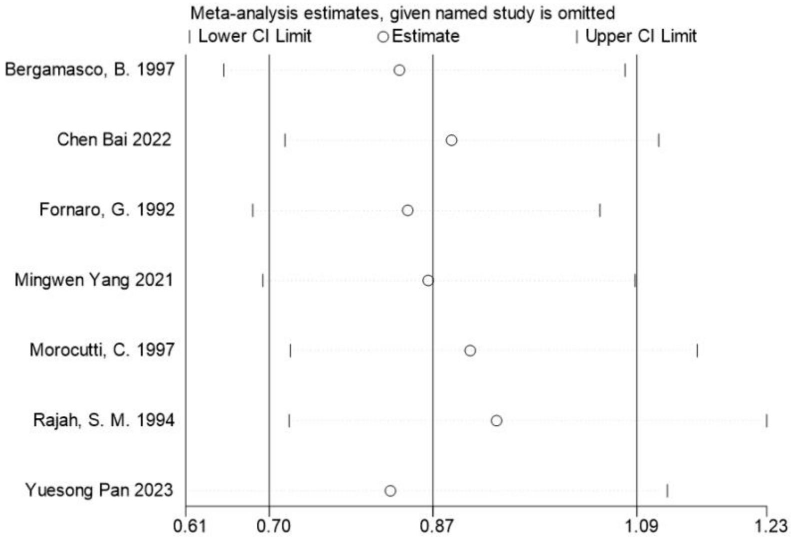

Supplementary Figure 9. Sensitivity analysis of total adverse reaction events.

1.2 Funnel plots

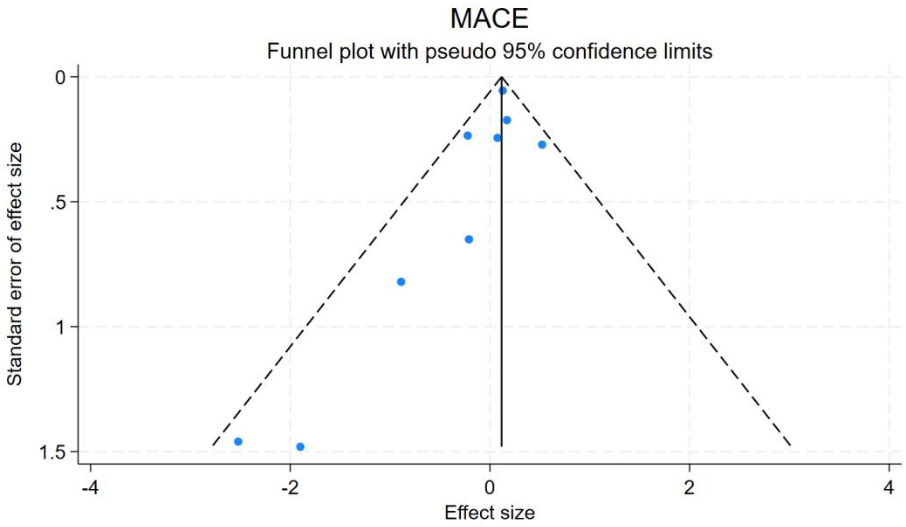

Supplementary Figure 10. Funnel plots of major adverse cardiovascular events (MACE).

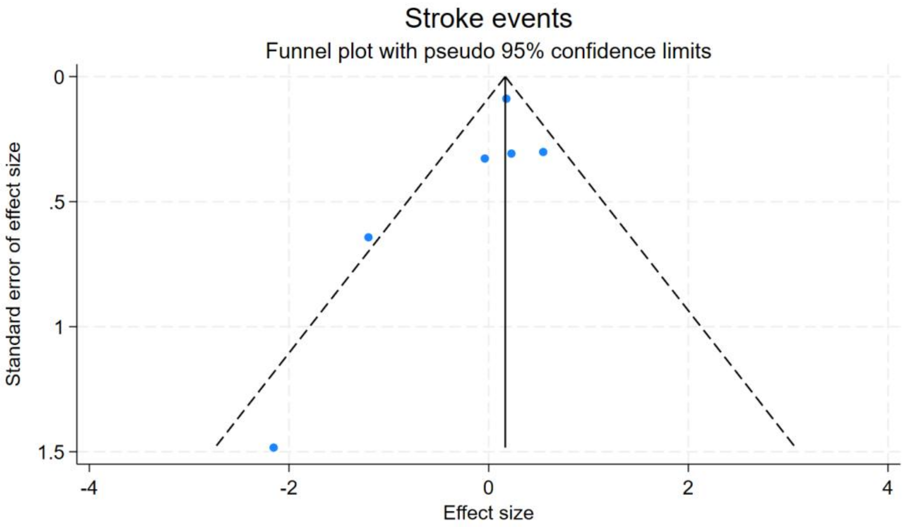

Supplementary Figure 11. Funnel plots of stroke events.

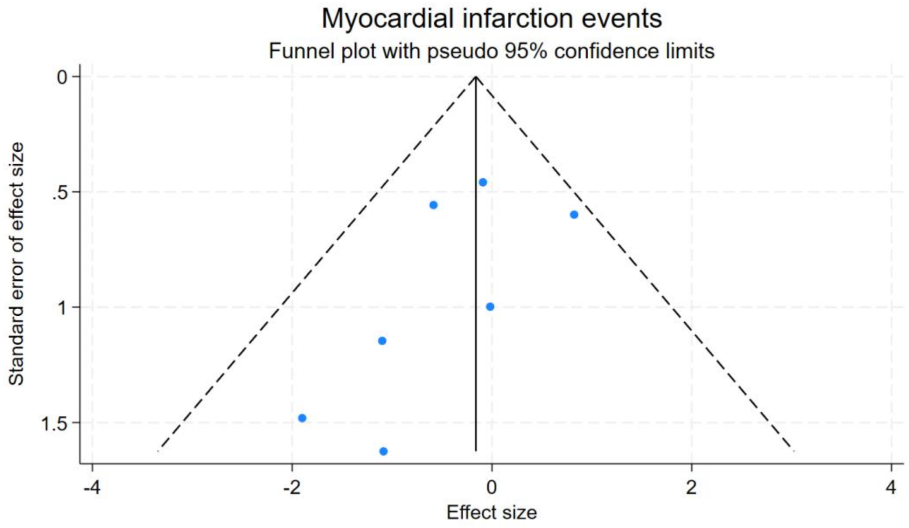

Supplementary Figure 12. Funnel plots of myocardial infarction events.

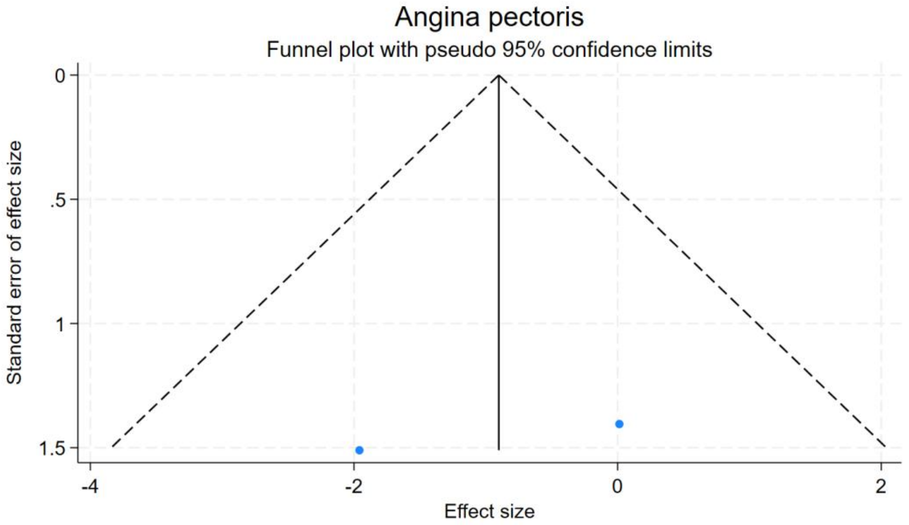

Supplementary Figure 13. Funnel plots of recurrent angina events.

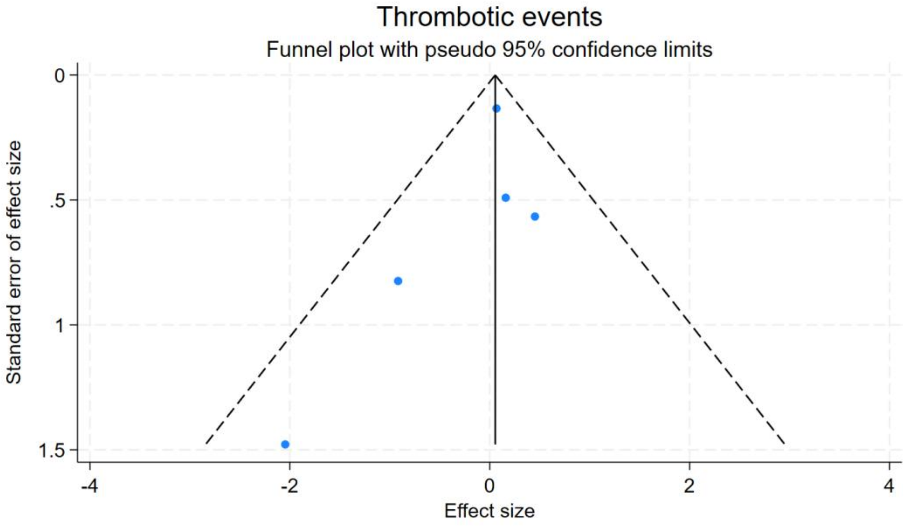

Supplementary Figure 14. Funnel plots of thrombotic events.

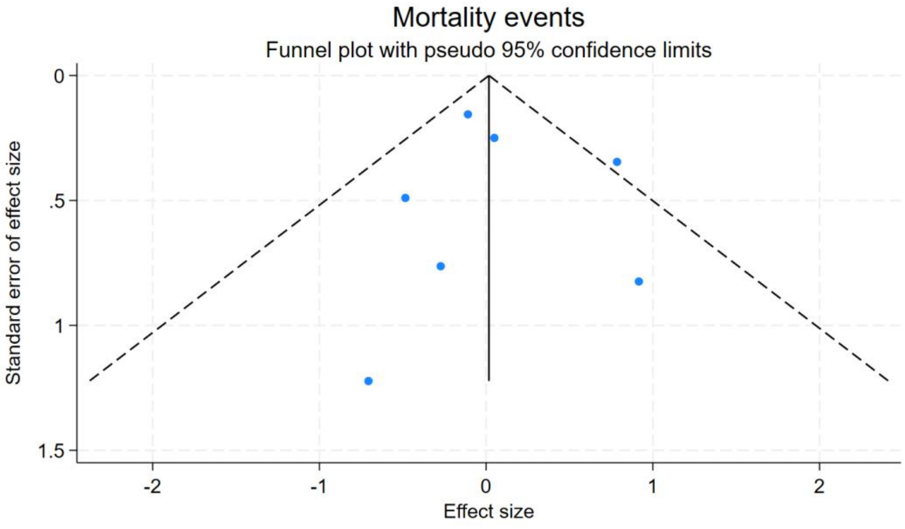

Supplementary Figure 15. Funnel plots of death outcomes.

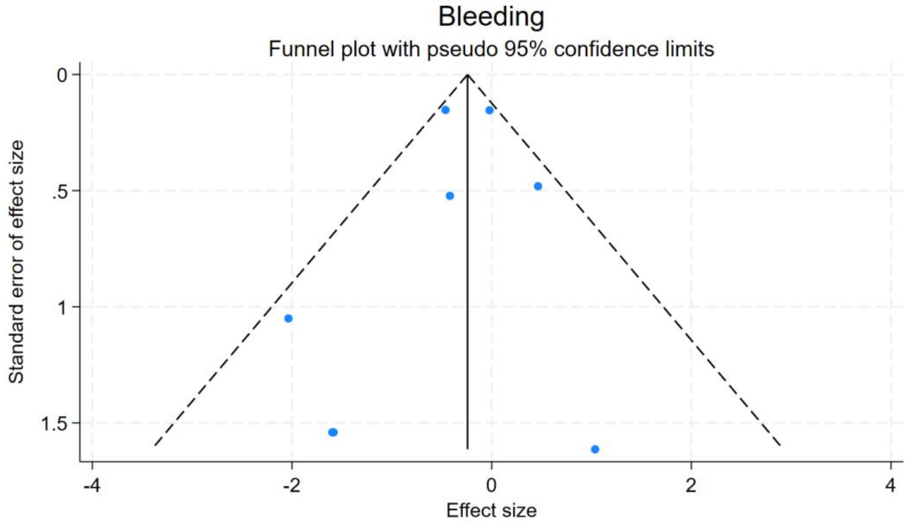

Supplementary Figure 16. Funnel plots of bleeding events.

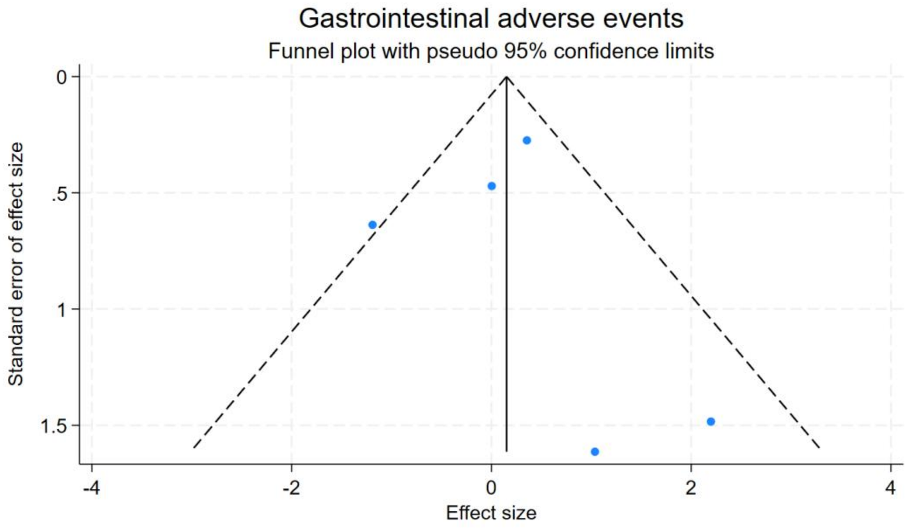

Supplementary Figure 17. Funnel plots of gastrointestinal adverse events.

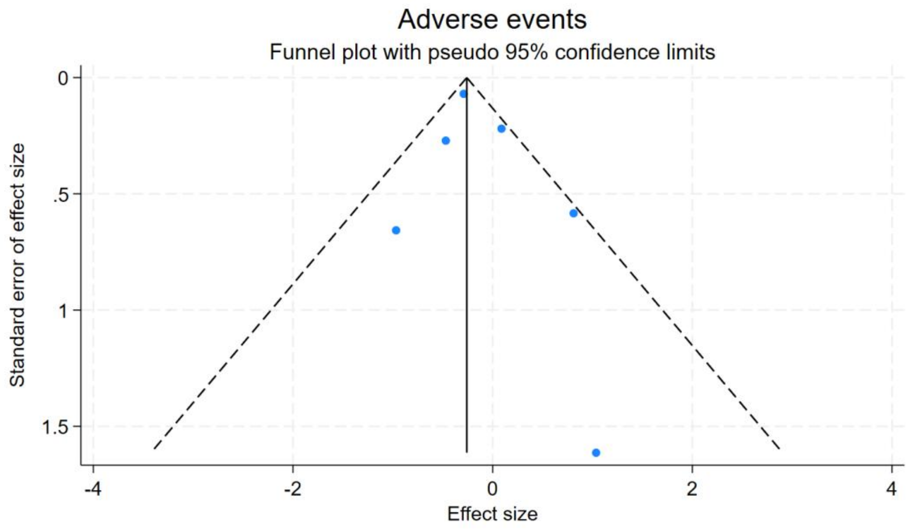

Supplementary Figure 18. Funnel plots of adverse events.

1.3 Forest plots of disease type subgroup analysis

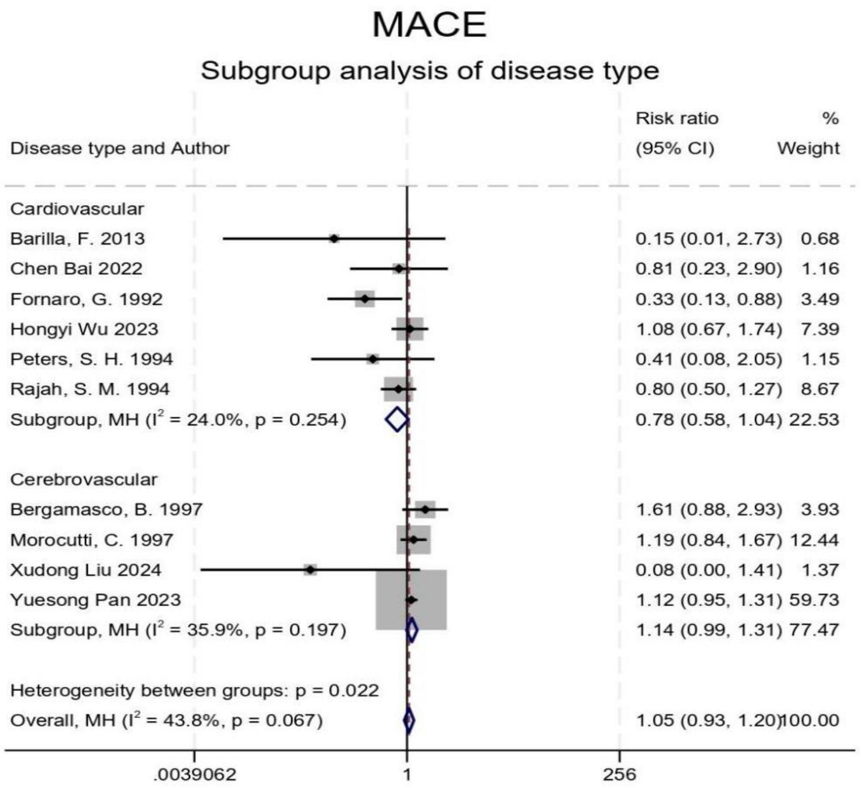

Supplementary Figure 19. Forest plot of MACE events subgroup analysis.

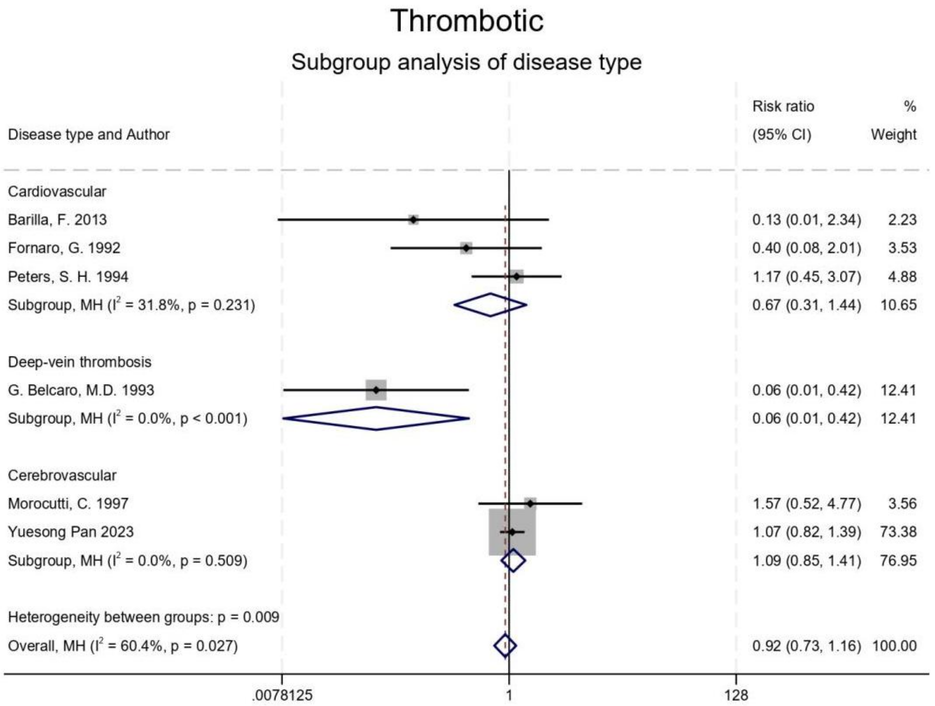

Supplementary Figure 20. Forest plot of thrombotic events subgroup analysis.

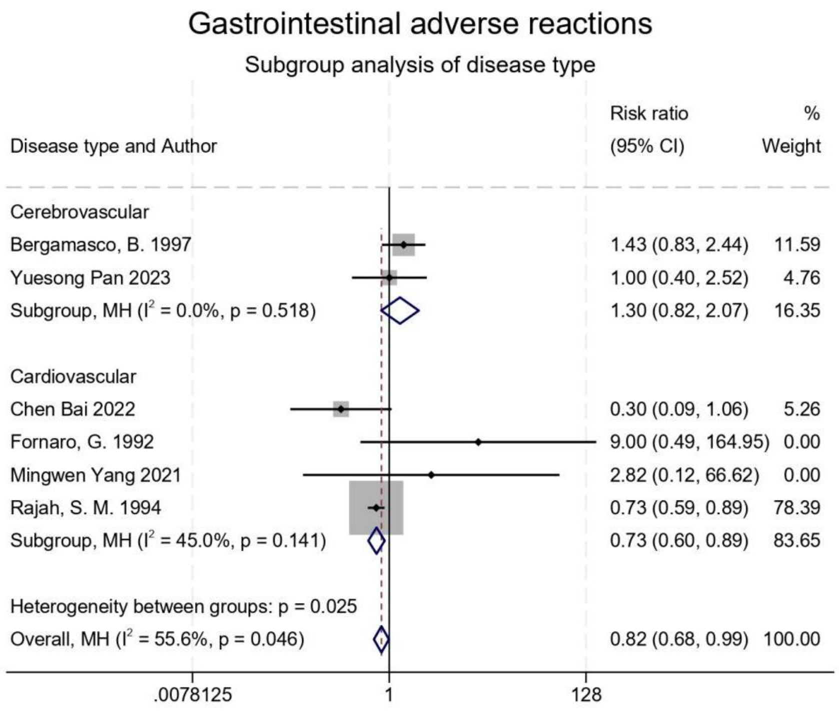

Supplementary Figure 21. Forest plot of gastrointestinal adverse events subgroup analysis.

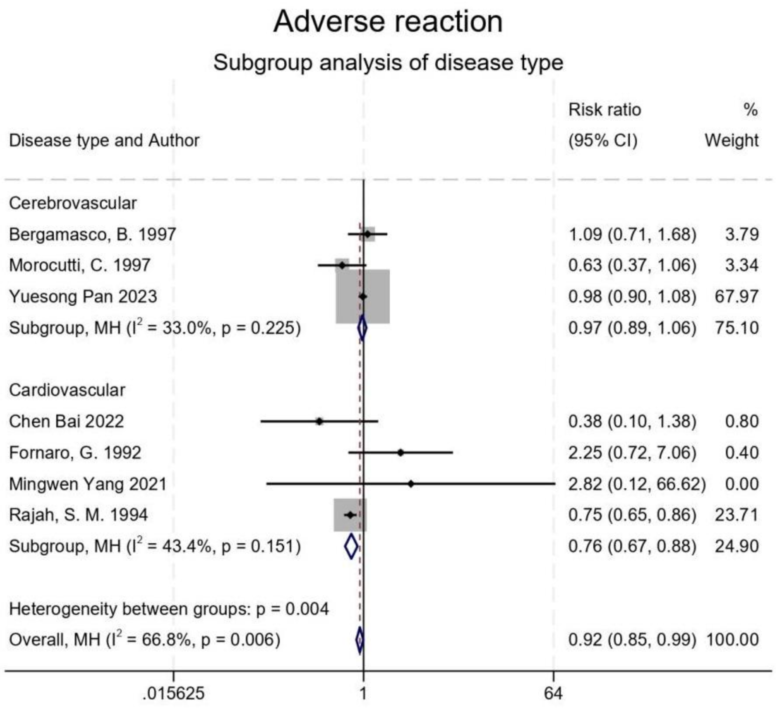

Supplementary Figure 22. Forest plot of adverse events subgroup analysis.

2 Supplementary Tables

Supplementary Table 2.1 Subgroup analysis of disease type.

	Cardiovascular				Cerebrovascular			
	RR	95% CI	I^2	P	RR	95% CI	I^2	P
MACE	0.78	0.58-1.04	24%	0.09	1.14	0.99-1.31	36%	0.07
Stroke	0.61	0.20-1.87	62%	0.39	1.22	1.04-1.43	23%	0.02
Myocardial infarction	0.62	0.29-1.31	0%	0.21	1.07	0.53-2.16	33%	0.85
Thrombotic	0.68	0.32-1.44	31%	0.31	1.09	0.85-1.41	0%	0.49
Death	0.85	0.43-1.65	0%	0.62	1.18	0.74-1.86	64%	0.49
Bleeding	0.62	0.47-0.83	0%	0.001	0.5	0.17-1.49	81%	0.21
Gastrointestinal adverse reactions	0.73	0.60-0.90	45%	0.002	1.30	0.82-2.07	0%	0.26
Adverse reactions	0.76	0.67-0.88	43%	0.0001	0.97	0.89-1.06	33%	0.55

Supplementary Table 2.2 Subgroup analysis of the region.

	Asian countries				Non-Asian countries			
	RR	95% CI	I^2	P	RR	95% CI	I^2	P
MACE	1.09	0.94-1.26	15%	0.27	0.48	0.13-1.34	82%	0.29
Stroke	1.16	0.98-1.37	30%	0.09	1.05	0.49-2.24	67%	0.9
Myocardial infarction	0.71	0.37-1.40	0%	0.33	1.01	0.46-2.21	32%	0.98
Thrombotic	1.07	0.82-1.39		0.61	0.48	0.14-1.58	68%	0.22
Death	0.89	0.66-1.20	0%	0.45	1.25	0.89-1.77	39%	0.20
Bleeding	0.72	0.45-1.14	53%	0.16	0.48	0.14-1.69	74%	0.25
Gastrointestinal adverse reactions	0.69	0.35-1.38	34%	0.3	1.11	0.53-2.33	76%	0.77
Adverse reactions	0.98	0.89-1.07	20%	0.64	0.86	0.62-1.20	56%	0.37

Supplementary Table 2.3 Subgroup analysis of drug type.

	Indobufen vs. Asprin / Clopidogrel				Indobufen vs. Others			
	RR	95% CI	I^2	P	RR	95% CI	I^2	P
MACE	1.05	0.91-1，21	28%	0.52	0.89	0.47-1.66	67%	0.71
Stroke	1.15	0.97-1.36	40%	0.1	1.05	0.49-2.24	67%	0.9
Myocardial infarction	0.63	0.33-1.21	0%	0.16	1.34	0.57-3.16	15%	0.51
Thrombotic	1.04	0.80-1.35	51%	0.78	0.56	0.16-2.01	72%	0.37
Death	0.85	0.43-1.65	0%	0.62	1.18	0.74-1.86	64%	0.49
Bleeding	0.75	0.61-0.92	41%	0.006	0.39	0.05-2.91	82%	0.36
Gastrointestinal adverse reactions	0.72	0.59-0.88	1%	0.001	1.6	0.95-2.69	34%	0.08
Adverse reactions	0.84	0.64-1.10	77%	0.2	1	0.56-1.76	60%	0.99

Supplementary Table 2.4 Subgroup analysis of study type.

	Unblinded				Double-blind			
	RR	95% CI	I²	P	RR	95% CI	I²	P
MACE	1.11	0.87-1.41	28%	0.39	0.77	0.47-1.24	65%	0.28
Stroke	1.21	0.86-1.71	32%	0.27	0.69	0.18-2.61	78%	0.59
Myocardial infarction	1.01	0.50-1.85	0%	0.96	0.50	0.19-1.33	0%	0.17
Thrombotic	0.26	0.02-3.31	81%	0.3	1.05	0.82-1.34	0%	0.71
Death	1.33	0.91-1.94	43%	0.14	0.89	0.67-1.18	0%	0.43
Bleeding	0.48	0.20-1.15	65%	0.1	0.93	0.70-1.24	0%	0.61
Gastrointestinal adverse reactions	0.89	0.26-3.04	62%	0.85	0.77	0.63-0.94	41%	0.01
Adverse reactions	0.84	0.61-1.15	35	0.27	0.91	0.69-1.20	85%	0.5

3 Database search strategy

3.1 Pubmed search strategy

(((((("Cerebrovascular Disorders"[Mesh]) OR ((Cerebrovascular Disorders[Title/Abstract]) OR (Cerebrovascular Disorder[Title/Abstract]) OR (Brain Vascular Disorders[Title/Abstract]) OR (Brain Vascular Disorder[Title/Abstract]) OR (Vascular Disorder, Brain[Title/Abstract]) OR (Vascular Disorders, Brain[Title/Abstract]) OR (Intracranial Vascular Disorders[Title/Abstract]) OR (Intracranial Vascular Disorder[Title/Abstract]) OR (Vascular Disorder, Intracranial[Title/Abstract]) OR (Vascular Disorders, Intracranial[Title/Abstract]) OR (Vascular Diseases, Intracranial[Title/Abstract]) OR (Intracranial Vascular Disease[Title/Abstract]) OR (Intracranial Vascular Diseases[Title/Abstract]) OR (Vascular Disease, Intracranial[Title/Abstract]) OR (Cerebrovascular Diseases[Title/Abstract]) OR (Cerebrovascular Disease[Title/Abstract]) OR (Disease, Cerebrovascular[Title/Abstract]) OR (Diseases, Cerebrovascular[Title/Abstract]) OR (Cerebrovascular Insufficiency[Title/Abstract]) OR (Cerebrovascular Insufficiencies[Title/Abstract]) OR (Insufficiencies, Cerebrovascular[Title/Abstract]) OR (Insufficiency, Cerebrovascular[Title/Abstract]) OR (Cerebrovascular Occlusion[Title/Abstract]) OR (Cerebrovascular Occlusions[Title/Abstract]) OR (Occlusion, Cerebrovascular[Title/Abstract]) OR (Occlusions, Cerebrovascular[Title/Abstract]))) OR ("Cardiovascular Diseases"[Mesh])) OR ((Cardiovascular Diseases[Title/Abstract]) OR (Cardiovascular Disease[Title/Abstract]) OR (Disease, Cardiovascular[Title/Abstract]) OR (Cardiac Events[Title/Abstract]) OR (Cardiac Event[Title/Abstract]) OR (Event, Cardiac[Title/Abstract]) OR (Adverse Cardiac Event[Title/Abstract]) OR (Adverse Cardiac Events[Title/Abstract]) OR (Cardiac Event, Adverse[Title/Abstract]) OR (Cardiac Events, Adverse[Title/Abstract]) OR (Major Adverse Cardiac Events[Title/Abstract]))) OR ("Thromboembolism"[Mesh])) OR ((thromboembolic disease[Title/Abstract]) OR (Thromboembolisms[Title/Abstract]) OR (Thromboembolism[Title/Abstract]))) AND (("indobufen" [Supplementary Concept]) OR ((indobufen[Title/Abstract]) OR (2-(4-(1-oxo-2-isoindolinyl)phenyl)butyric acid[Title/Abstract]) OR (K 3920[Title/Abstract]) OR (Ibustrin[Title/Abstract]) OR (indobufen, (+-)-isomer[Title/Abstract])))) AND (("Randomized Controlled Trial" [Publication Type]) OR ((randomized controlled trial[Title/Abstract]) OR (randomized[Title/Abstract]) OR (placebo[Title/Abstract])))

3.2 Embase search strategy

No.	Query Results	Results	Date
#13.	#8 AND #9 AND #12	80	2 Sep 2024
#12.	#10 OR #11	575	2 Sep 2024
#11.	'2-(4-(1-oxo-2-isoindolinyl)phenyl)butyric acid':ab,ti OR 'indobufen, (+-)-isomer':ab,ti OR '1 oxo 2 [4 [(alpha ethyl) carboxymethyl] phenyl] isoindoline':ab,ti OR '2 [4 (1 oxo 2 isoindolinyl) phenyl] butanoic acid':ab,ti OR '2	244	2 Sep 2024

	[4 (1 oxo 2 isoindolinyl) phenyl] butyric acid':ab,ti OR 'alpha [4 (1 oxo 2 isoindolinyl) phenyl] butyric acid':ab,ti OR 'ibustrin':ab,ti OR 'k 3920':ab,ti OR 'indobufen':ab,ti		
#10.	'indobufen'/exp	564	2 Sep 2024
#9.	'randomized controlled trial':ab,ti OR 'randomized':ab,ti OR 'placebo':ab,ti	1,211,970	2 Sep 2024
#8.	#1 OR #2 OR #3 OR #4 OR #5 OR #6 OR #7	5,820,517	2 Sep 2024
#7.	'cardiovascular and cerebrovascular diseases':ab,ti	1,929	2 Sep 2024
#6.	'cerebral embolism and thrombosis':ab,ti OR 'embolism and thrombosis':ab,ti OR 'embolism, thrombo':ab,ti OR 'intracranial embolism and thrombosis':ab,ti OR 'thrombo embolic disease':ab,ti OR 'thrombo embolism':ab,ti OR 'thrombo-emboli':ab,ti OR 'thrombo-embolus':ab,ti OR 'thromboemboli':ab,ti OR 'thromboembolic':ab,ti OR 'thromboembolic complication':ab,ti OR 'thromboembolic disease':ab,ti OR 'thromboembolic process':ab,ti OR 'thromboembolus':ab,ti OR 'thromboemboly':ab,ti OR 'thromboembolism':ab,ti	129,201	2 Sep 2024
#5.	'thromboembolism'/exp	707,781	2 Sep 2024
#4.	'cardiac events':ab,ti OR 'cardiac event':ab,ti OR 'event, cardiac':ab,ti OR 'adverse cardiac	385,426	2 Sep 2024

event':ab,ti OR 'adverse cardiac events':ab,ti OR 'cardiac event, adverse':ab,ti OR 'cardiac events, adverse':ab,ti OR 'major adverse cardiac events':ab,ti OR 'angiocardiopathy':ab,ti OR 'angiocardiovascular disease':ab,ti OR 'cardiovascular complication':ab,ti OR 'cardiovascular diseases':ab,ti OR 'cardiovascular disorder':ab,ti OR 'cardiovascular disturbance':ab,ti OR 'cardiovascular lesion':ab,ti OR 'cardiovascular syndrome':ab,ti OR 'cardiovascular vegetative disorder':ab,ti OR 'complication, cardiovascular':ab,ti OR 'disease, cardiovascular':ab,ti OR 'major adverse cardiovascular event':ab,ti OR 'cardiovascular disease':ab,ti

#3. 'cardiovascular disease'/exp 5,748,250 2 Sep 2024

#2. 'brain vascular disorders':ab,ti OR 'brain vascular disorder':ab,ti OR 'vascular disorder, brain':ab,ti OR 'vascular disorders, brain':ab,ti OR 'intracranial vascular disorders':ab,ti OR 'intracranial vascular disorder':ab,ti OR 'vascular disorder, intracranial':ab,ti OR 'vascular disorders, intracranial':ab,ti OR 'vascular diseases, intracranial':ab,ti OR 'intracranial vascular disease':ab,ti OR 155,866 2 Sep 2024

'intracranial vascular diseases':ab,ti OR 'vascular disease, intracranial':ab,ti OR 'cerebrovascular diseases':ab,ti OR 'disease, cerebrovascular':ab,ti OR 'diseases, cerebrovascular':ab,ti OR 'cerebrovascular insufficiency':ab,ti OR 'cerebrovascular insufficiencies':ab,ti OR 'insufficiencies, cerebrovascular':ab,ti OR 'insufficiency, cerebrovascular':ab,ti OR 'cerebrovascular occlusion':ab,ti OR 'cerebrovascular occlusions':ab,ti OR 'occlusion, cerebrovascular':ab,ti OR 'occlusions, cerebrovascular':ab,ti OR 'cardiovascular diseases':ab,ti OR 'brain angiopathy':ab,ti OR 'brain circulation failure':ab,ti OR 'brain vascular disease':ab,ti OR 'brain vasculopathy':ab,ti OR 'cerebral angiopathy':ab,ti OR 'cerebral small vessel disease':ab,ti OR 'cerebral small vessel diseases':ab,ti OR 'cerebral vascular disease':ab,ti OR 'cerebral vascular disorder':ab,ti OR 'cerebral vascular disturbance':ab,ti OR 'cerebral vascular lesion':ab,ti OR 'cerebral vasculopathy':ab,ti OR 'cerebro-vascular damage':ab,ti OR

'cerebro-vascular disease':ab,ti OR

'cerebro-vascular disorder':ab,ti OR

'cerebro-vascular disturbance':ab,ti OR

'cerebro-vascular lesion':ab,ti OR

'cerebro-vascular pathology':ab,ti OR

'cerebro-vascular syndrome':ab,ti OR

'cerebro-vasculopathy':ab,ti OR

'cerebroangiopathy':ab,ti OR 'cerebrovascular damage':ab,ti OR 'cerebrovascular disorder':ab,ti OR 'cerebrovascular disorders':ab,ti OR

'cerebrovascular disturbance':ab,ti OR

'cerebrovascular lesion':ab,ti OR

'cerebrovascular pathology':ab,ti OR

'cerebrovascular syndrome':ab,ti OR

'cerebrovasculopathy':ab,ti OR 'cerebrovascular disease':ab,ti

#1. 'cerebrovascular disease'/exp 974,709 2 Sep 2024

3.3 The Cochrane Library search strategy

Search Name:

Date Run: 02/09/2024 11:44:59

Comment:

ID Search Hits

#1 MeSH descriptor: [Cerebrovascular Disorders] explode all trees 25536

#2 (Cerebrovascular Disorder):ti,ab,kw OR (Brain Vascular Disorders):ti,ab,kw OR (Brain Vascular Disorder):ti,ab,kw OR (Vascular Disorder, Brain):ti,ab,kw OR (Vascular Disorders, Brain):ti,ab,kw OR (Intracranial Vascular Disorders):ti,ab,kw OR (Intracranial Vascular

Disorder):ti,ab,kw OR (Vascular Disorder, Intracranial):ti,ab,kw OR (Vascular Disorders, Intracranial):ti,ab,kw OR (Vascular Diseases, Intracranial):ti,ab,kw OR (Intracranial Vascular Disease):ti,ab,kw OR (Intracranial Vascular Diseases):ti,ab,kw OR (Vascular Disease, Intracranial):ti,ab,kw OR (Cerebrovascular Diseases):ti,ab,kw OR (Cerebrovascular Disease):ti,ab,kw OR (Disease, Cerebrovascular):ti,ab,kw OR (Diseases, Cerebrovascular):ti,ab,kw OR (Cerebrovascular Insufficiency):ti,ab,kw OR (Cerebrovascular Insufficiencies):ti,ab,kw OR (Insufficiencies, Cerebrovascular):ti,ab,kw OR (Insufficiency, Cerebrovascular):ti,ab,kw OR (Cerebrovascular Occlusion):ti,ab,kw OR (Cerebrovascular Occlusions):ti,ab,kw OR (Occlusion, Cerebrovascular):ti,ab,kw OR (Occlusions, Cerebrovascular):ti,ab,kw 16620

#3 MeSH descriptor: [Cardiovascular Diseases] explode all trees 157319

#4 (Cardiovascular Disease):ti,ab,kw OR (Disease, Cardiovascular):ti,ab,kw OR (Cardiac Events):ti,ab,kw OR (Cardiac Event):ti,ab,kw OR (Event, Cardiac):ti,ab,kw OR (Adverse Cardiac Event):ti,ab,kw OR (Adverse Cardiac Events):ti,ab,kw OR (Cardiac Event, Adverse):ti,ab,kw OR (Cardiac Events, Adverse):ti,ab,kw OR (Major Adverse Cardiac Events):ti,ab,kw 67654

#5 (thromboembolic disease):ti,ab,kw OR (Thromboembolisms):ti,ab,kw 2072

#6 (Cardiovascular and cerebrovascular diseases):ti,ab,kw 1908

#7 #1 OR #2 OR #3 OR #4 # OR #5 OR #6 204745

#8 (indobufen):ti,ab,kw OR (K 3920):ti,ab,kw OR (Ibustrin):ti,ab,kw OR (indobufen,isomer):ti,ab,kw 118

#9 (randomized controlled trial):ti,ab,kw OR (randomized):ti,ab,kw OR (placebo):ti,ab,kw 1323347

#10 #7 AND #8 AND #9 44

3.4 Web of Science search strategy

1: TI=(Cerebrovascular Disorders) OR TI=(Cerebrovascular Disorder) OR TI=(Brain Vascular Disorders) OR TI=(Brain Vascular Disorder) OR TI=(Vascular Disorder, Brain) OR TI=(Vascular Disorders, Brain) OR TI=(Intracranial Vascular Disorders) OR TI=(Intracranial Vascular Disorder) OR TI=(Vascular Disorder, Intracranial) OR TI=(Vascular Disorders, Intracranial) OR TI=(Vascular Diseases, Intracranial) OR TI=(Intracranial Vascular Disease) OR TI=(Intracranial Vascular Diseases) OR TI=(Vascular Disease, Intracranial) OR TI=(Cerebrovascular Diseases) OR TI=(Cerebrovascular Disease) OR TI=(Disease, Cerebrovascular) OR TI=(Diseases, Cerebrovascular) OR TI=(Cerebrovascular Insufficiency) OR TI=(Cerebrovascular Insufficiencies) OR TI=(Insufficiencies, Cerebrovascular) OR TI=(Insufficiency, Cerebrovascular) OR TI=(Cerebrovascular Occlusion) OR TI=(Cerebrovascular Occlusions) OR TI=(Occlusion, Cerebrovascular) OR TI=(Occlusions, Cerebrovascular)

Date: Mon Sep 02 2024 21:19:56 GMT+0800 (CST) search result: 8263

2: TI=(Cardiovascular Diseases) OR TI=(Cardiovascular Disease) OR TI=(Disease, Cardiovascular) OR TI=(Cardiac Events) OR TI=(Cardiac Event) OR TI=(Event, Cardiac) OR TI=(Adverse Cardiac

Event) OR TI=(Adverse Cardiac Events) OR TI=(Cardiac Event, Adverse) OR TI=(Cardiac Events, Adverse) OR TI=(Major Adverse Cardiac Events)

Date: Mon Sep 02 2024 21:21:34 GMT+0800 (CST) search result: 76912

3: TI=(thromboembolic disease) OR TI=(Thromboembolisms)

Date: Mon Sep 02 2024 21:24:00 GMT+0800 (CST) search result: 2292

4: #1 OR #2 OR #3

Date: Mon Sep 02 2024 21:24:26 GMT+0800 (CST) search result: 86998

5: TI=(indobufen) OR TI=(2-(4-(1-oxo-2-isoindolinyl)phenyl)butyric acid) OR TI=(K 3920) OR TI=(Ibustrin) OR TI=(indobufen, (+-)-isomer)

Date: Mon Sep 02 2024 21:25:09 GMT+0800 (CST) search result: 173

6: AB=(randomized controlled trial) OR AB=(randomized) OR AB=(placebo)

Date: Mon Sep 02 2024 21:25:27 GMT+0800 (CST) search result: 923533

7: #4 AND #5 AND #6

Date: Mon Sep 02 2024 21:30:14 GMT+0800 (CST) search result: 2
